# Supplementary material for: ROS-induced R loops trigger a transcription-coupled but BRCA1/2-independent homologous recombination pathway through CSB
Source: Nat Commun. 2018 Oct 8;9:4115. doi: 10.1038/s41467-018-06586-3 (PMC6175878; doi:10.1038/s41467-018-06586-3)
Supplement: Supplementary file 1 — Supplementary Information [file 41467_2018_6586_MOESM1_ESM.pdf]

ROS-Induced R Loops Trigger a Transcription-Coupled but BRCA1/2-Independent Homologous Recombination Pathway through CSB

Teng et.al

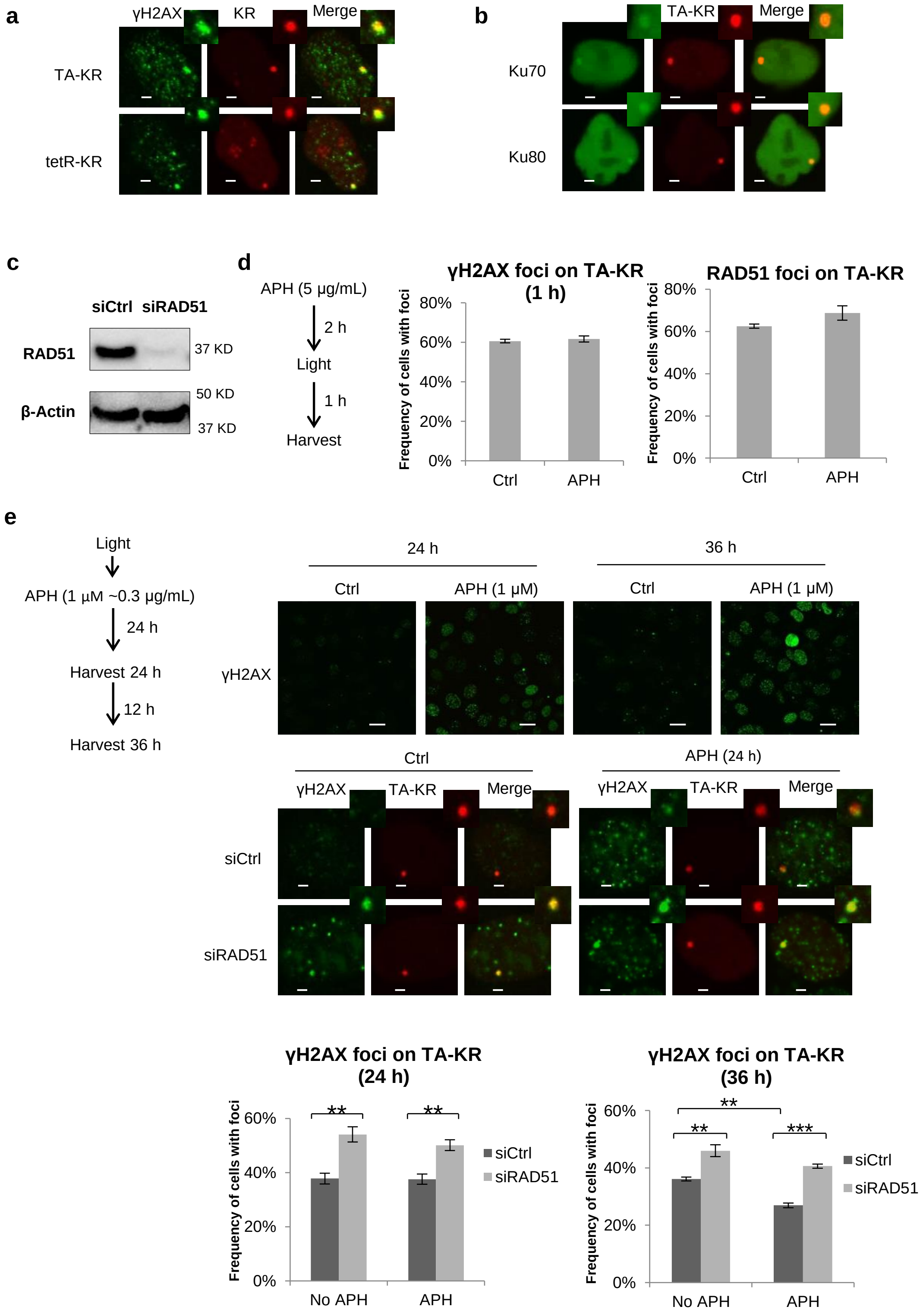

Supplementary Figure 1. RAD51 is required in TC-HR independent of replication.
(**a**) γH2AX foci are induced by TA-KR and tetR-KR upon light activation at a distinct genomic locus in U2OS TRE cells (scale bar: 2 μm). (**b**) Recruitment of GFP-tagged Ku70 and Ku80 to TA-KR in U2OS TRE cells (scale bar: 2 μm). (**c**) Western blot confirmation of siRAD51. (**d**) γH2AX foci and RAD51 foci frequency at TA-KR 1 h after light-induced KillerRed activation in cells treated with or without aphidicolin (APH, 5 μg/mL, 2 h). (**e**) γH2AX foci staining at TA-KR in control or RAD51 knockdown cells treated with or without APH (1 μM, ~0.3 μg/mL) for 24 h or 36 h after light-induced KillerRed activation (scale bar: upper panel, 20 μm; lower panel, 2 μm). For (d) and (e), n=3, 50 cells per replicate. Unpaired t-test, error bars represent SEM. *$P < 0.05$, **$P < 0.01$, ***$P < 0.001$.

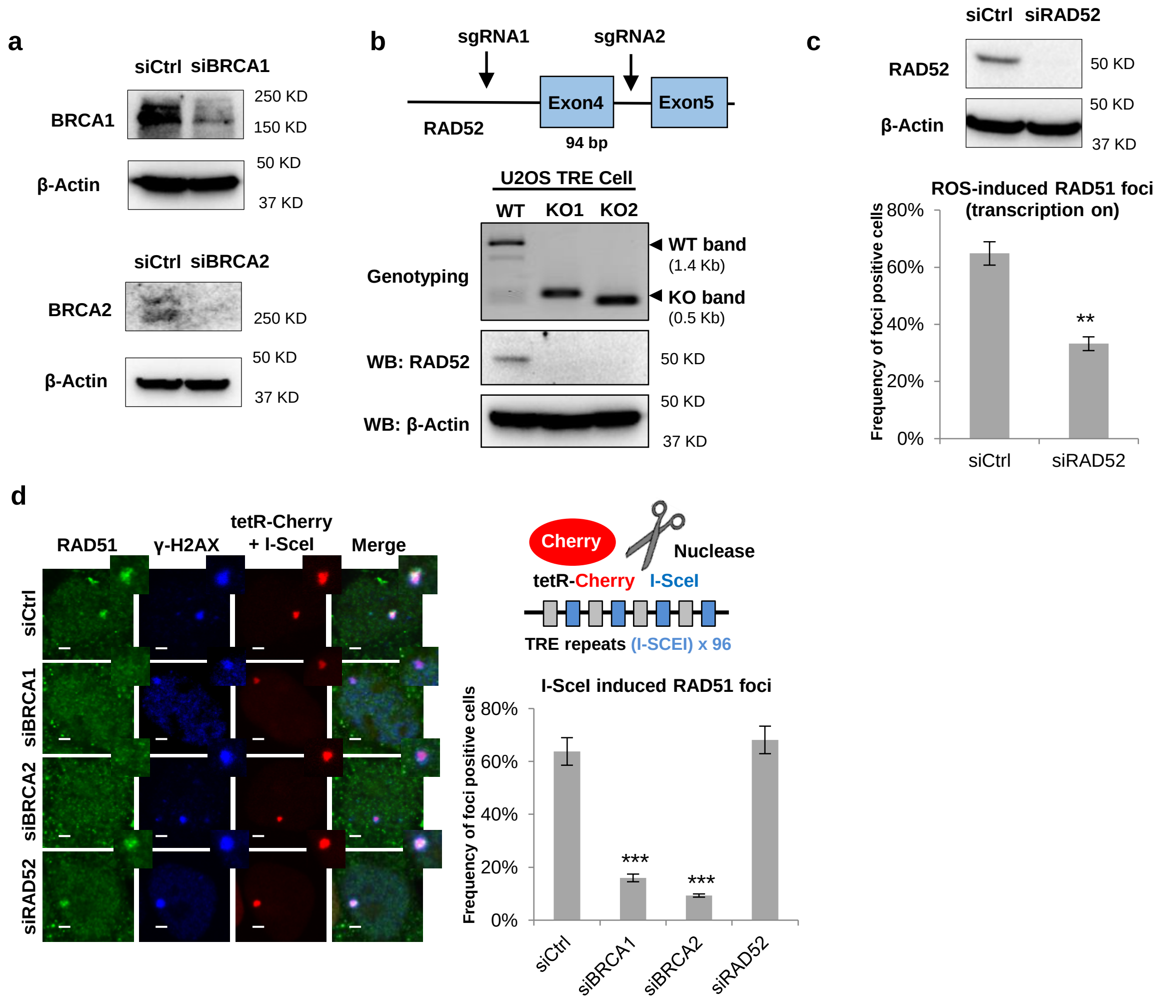

Supplementary Figure 2. RAD52 is required for RAD51 loading at ROS but not nuclease induced DSBs

(**a**) Western blot confirmation of siRAD51, siBRCA1, and siBRCA2. (**b**) RAD52 CRISPR-Cas9 KO design and confirmation by genotyping and Western blot. (**c**) Western blot of siRAD52, and RAD51 foci frequency at TA-KR in cells treated with control or RAD52 siRNAs. (**d**) RAD51 foci frequency at I-SceI endonuclease-induced damage sites marked by tetR-Cherry in U2OS TRE cells treated with control, BRCA1, BRCA2 or RAD52 siRNAs (scale bar: 2 μm). For (c) and (d), n=3, 50 cells per replicate. Unpaired *t*-test, error bars represent SEM. *P < 0.05, **P < 0.01, ***P < 0.001.

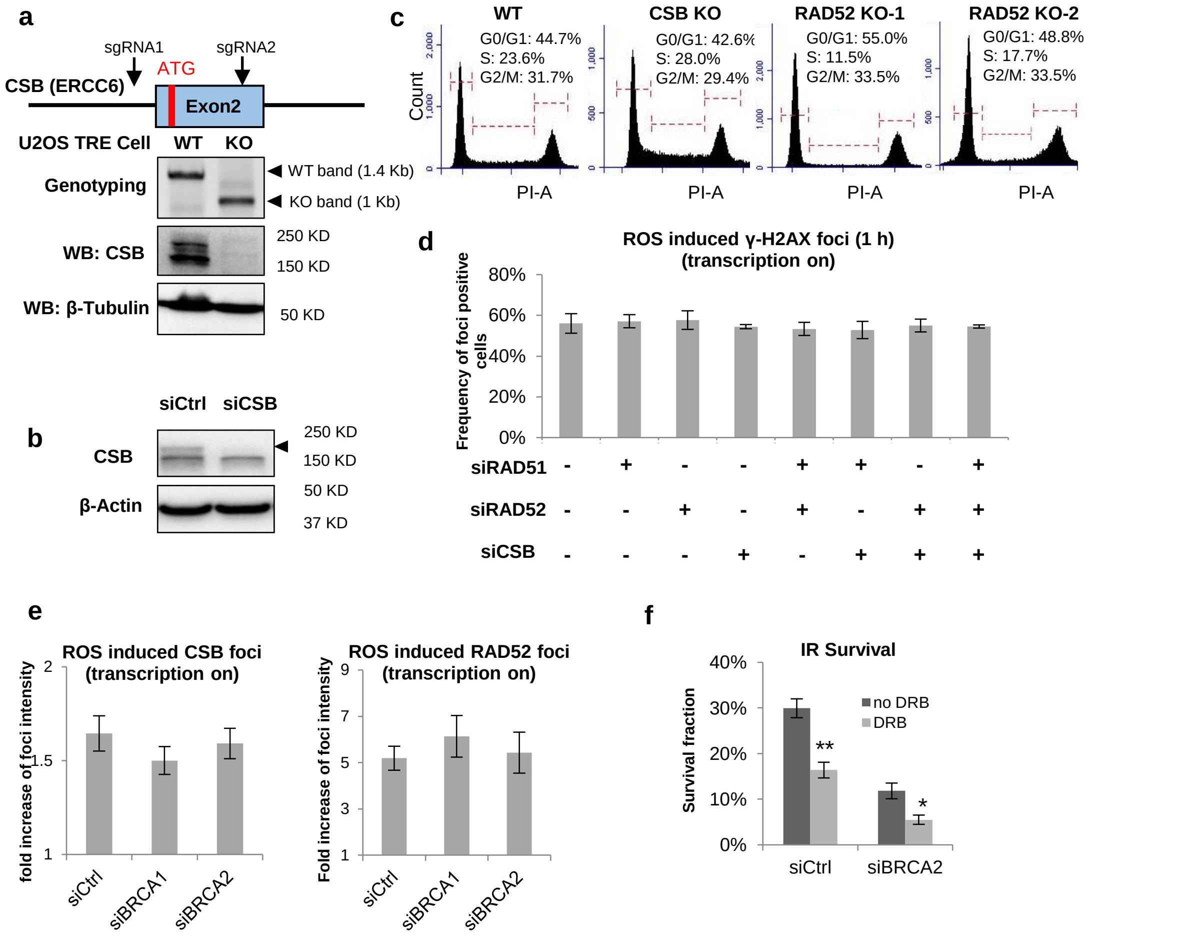

Supplementary Figure 3. CSB and RAD52 are recruited to ROS damage sites independently of BRCA2/1.

(**a**) CSB CRISPR-Cas9 KO design and confirmation by genotyping and Western blot. (**b**) Western blot confirmation of siCSB. (**c**) Flow cytometry analysis of U2OS TRE WT, CSB KO and RAD52 KO cell cycle profile by propidium iodide (PI) staining. (**d**) γH2AX foci frequency at TA-KR at an early (1 h) time point after damage induction in cells with single, double or triple knockdown of RAD51, RAD52 and CSB (n=3, 50 cells per replicate). (**e**) Damage response of CSB or RAD52 to TA-KR in siBRCA1 or siBRCA2 treated cells. The relative intensity of foci versus background is quantified (n=10 cells in one experiment). (**f**) Survival of siCtrl or siBRCA2 treated cells in a colony formation assay under 3 Gy IR irradiation and additional DRB (20 μM, 24 h) treatment (n=3). Unpaired *t*-test, error bars represent SEM. *P < 0.05, **P < 0.01, ***P < 0.001.

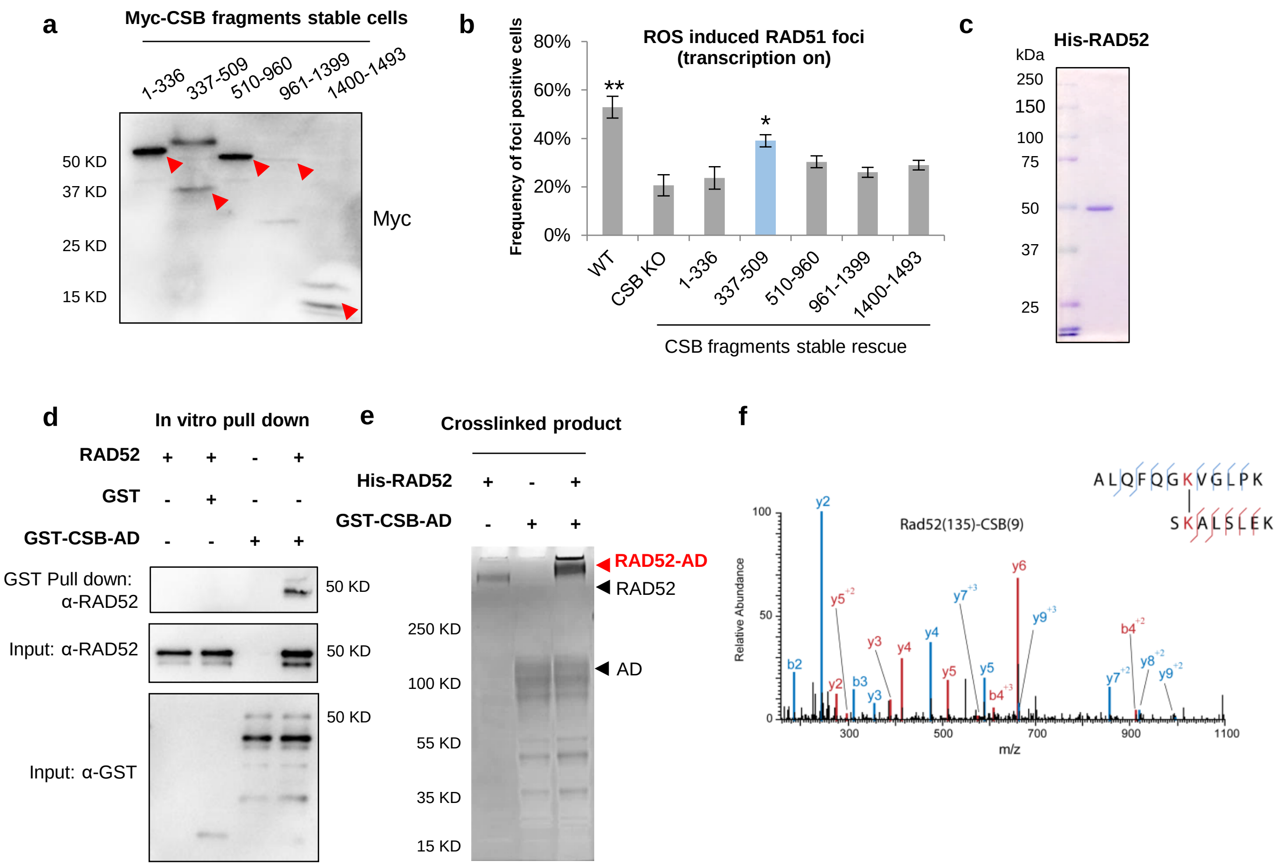

Supplementary Figure 4. CSB-AD binds to RAD52 *in vitro* and facilitates RAD51 damage response in cells. (**a**) Western blot confirmation of Myc-tagged CSB fragment stable expression cell lines constructed by lenti-virus infection of CSB KO cells. (**b**) RAD51 foci frequency at TA-KR in WT, CSB KO and CSB fragment stable expression cells. n=3, 50 cells per replicate. (**c**) SDS-PAGE of purified RAD52 protein. (**d**) Interaction of His-RAD52 and GST-CSB AD purified protein by anti-GST in an *in vitro* pull down. (**e**) SDS-PAGE of crosslink products of GST-CSB-AD, RAD52 protein and the combination of the two proteins. (**f**) A representative MS/MS crosslink spectrum between CSB AD and RAD52 protein is shown. Δm =1 ppm. Unpaired *t*-test, error bars represent SEM. *P < 0.05, **P < 0.01, ***P < 0.001.

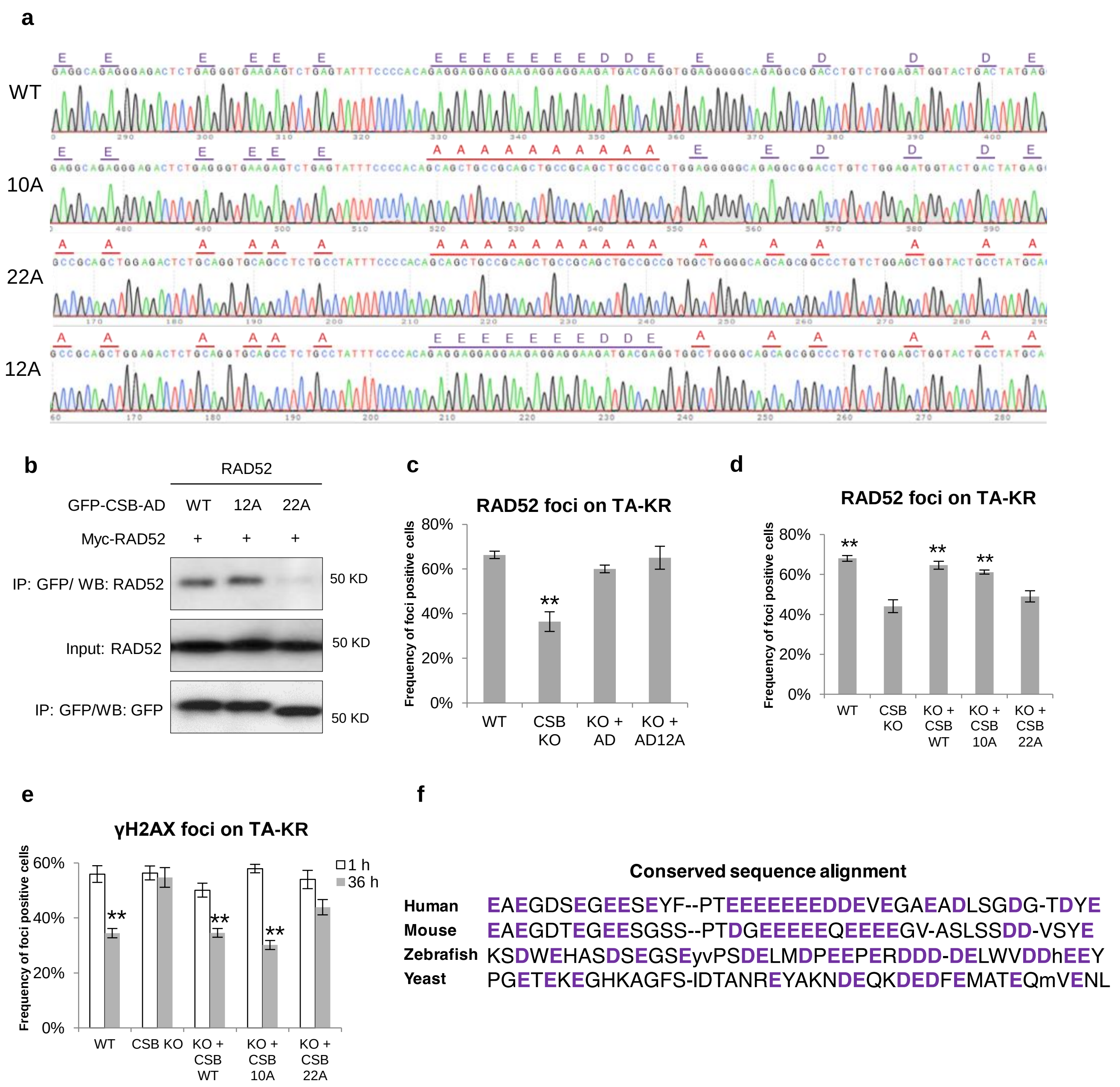

Supplementary Figure 5. The acidic residues are essential for the function of CSB-AD and CSB protein in TC-HR

(**a**) Sequencing confirmation of CSB-AD WT, 10A, 22A and 12A. The amino acids (E, D or A) were marked above the DNA codons. (**b**) Interactions between Myc-RAD52 and GFP-AD WT, 12A and 22A were tested by anti-GFP Co-IP in Flp-in 293 cells. (**c**) RAD52 foci frequency at TA-KR in WT, CSB KO cells and CSB KO cells transfected with GFP-AD or GFP-AD12A. (**d**) RAD52 foci frequency at TA-KR in WT, CSB KO cells and CSB KO cells transfected with Myc-CSB WT, 10A or 22A. (**e**) γH2AX foci frequency at TA-KR at early (1 h) and late (36 h) time points after damage in WT, CSB KO cells and CSB KO cells transfected with Myc-CSB WT, 10A or 22A. (**f**) Sequence alignment of the CSB-AD 22A region in human (Homo sapiens), mouse (Mus musculus), zebrafish (Danio rerio) and yeast (Saccharomyces cerevisiae). Acidic amino acids are marked in purple. For (c) to (e), n=3, 50 cells per replicate. Unpaired *t*-test, error bars represent SEM. *P < 0.05, **P < 0.01, ***P < 0.001.

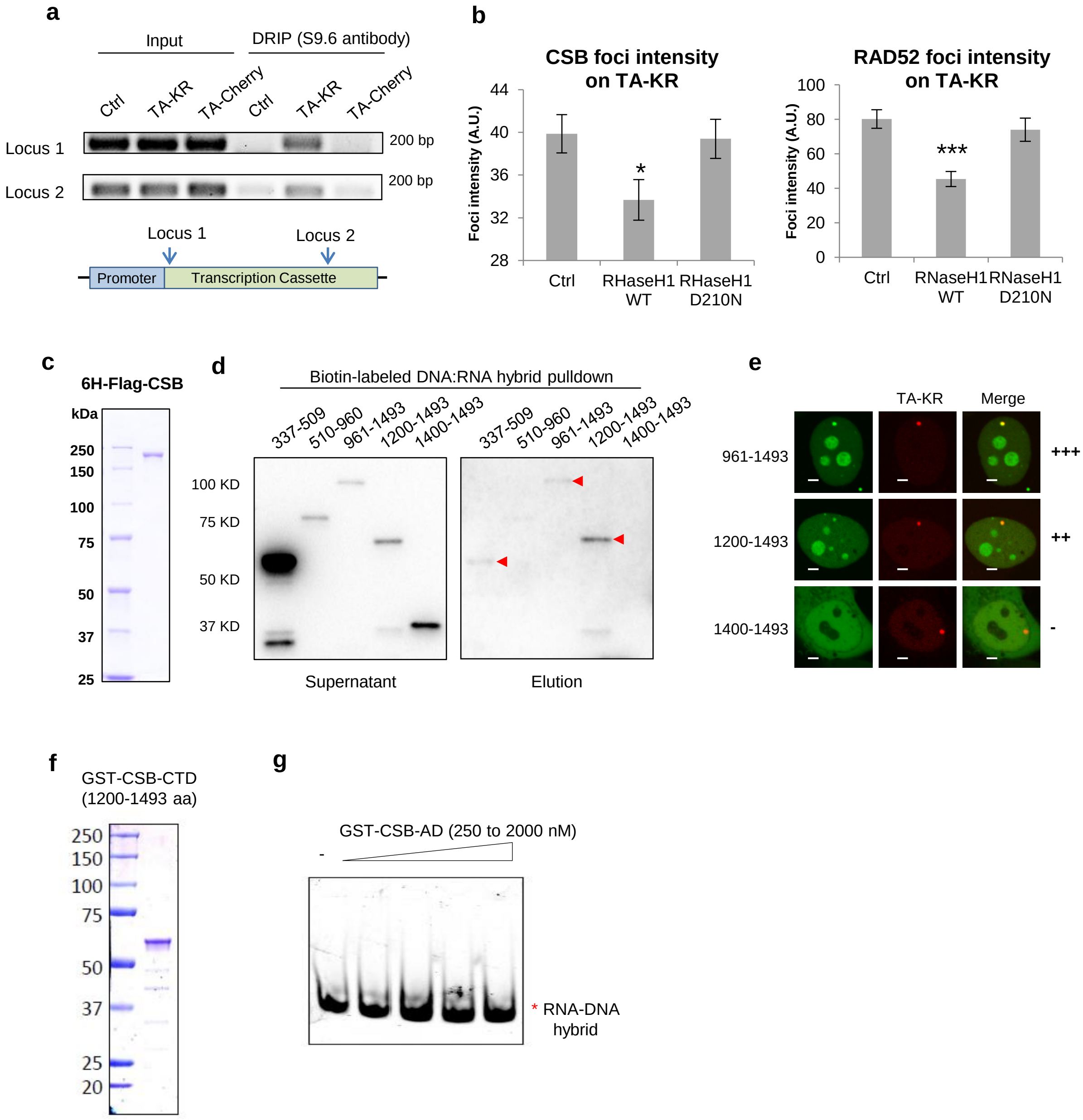

Supplementary Figure 6. CSB senses R loops through its C terminal region.

(a) DRIP-PCR detected DNA: RNA hybrids at two loci in transcription cassette near TRE region in cells transfected with TA-KR/TA-Cherry (related with Figure 4d). (b) Re-plotted figures of CSB, RAD52 foci using arbitrary intensity (A.U.) related with Figure 5b (n=10 cells in one experiment). (c) Coomassie staining of purified CSB protein. (d) Biotin-labeled DNA:RNA hybrid pulldown assay using cell lysates with GFP-CSB 337-509, 510-960, 961-1493, 1200-1493, 1400-1493 expression. (e) Recruitments of GFP-CSB 961-1493, 1200-1493 or 1400-1493 to TA-KR were tested and their relative foci intensity on TA-KR was marked on the side (- no foci, ++ strong foci, +++ stronger foci. scale bar: 2 μm). (f) Coomassie staining of purified GST-CSB-CTD (1200-1493) protein. (g) Purified GST-CSB-AD protein did not show binding to DNA:RNA hybrid in vitro by EMSA assay. Unpaired t-test, error bars represent SEM. *$P < 0.05$, **$P < 0.01$, ***$P < 0.001$.

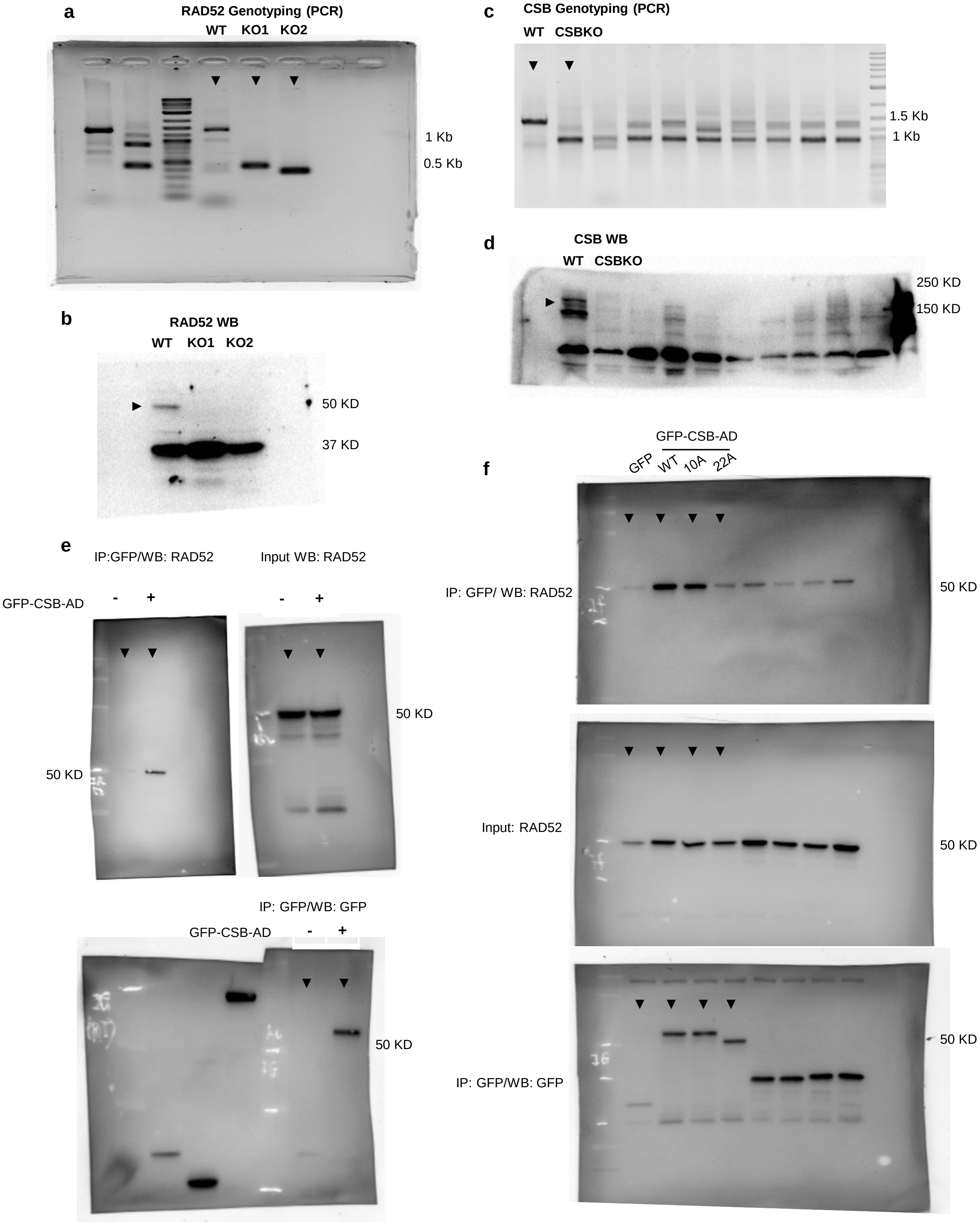

Supplementary Figure 7. Uncropped scans of important blots in this study.

(a) RAD52 genotyping PCR gel and (b) RAD52 western blot of WT and RAD52 KO cells (related with Supplemental Figure 2b). (c) CSB genotyping PCR gel and (d) CSB western blot of WT and CSB KO cells (related with Supplemental Figure 3a). (e) Co-IP between GFP-CSB-AD and Myc-RAD52 (related with Figure 3e). (f) Co-IP between GFP-CSB-AD mutants and Myc-RAD52 (related with Figure 3h).

Supplementary Table

Supplementary Table 1. Sequences of primers for sub-cloning

Oligo Name	Sequence (5'-3')
XhoI-CSB-1-F	CCGCTCGAGCCAAATGAGGGAATCC
CSB-336-NotI-R	ATAGTTTAGCGGCCGCCTGGAGTTTCTTGATGTG
XhoI-CSB-337-F:	CGGCTCGAGAAGAGGGCTTTGCAGTTCCAG
CSB-509-NotI-R	ATAGTTTAGCGGCCGCCTTAAAAAGCTTTTTGAACAG
XhoI-NLS-CSB-510-F	CCGCTCGAGCCTCCAAAAAAGAAGAGAAAGGTAGGCGGCGGCT ACCAGCAGACAGGTG
CSB-960-stop-NotI-R	ATAGTTTAGCGGCCGCTTAGTACACAGTCACTTGCTTC
XhoI-CSB-961-F	CCGCTCGAGAGGCTCCTGACTGCG
CSB-1399-NotI-R	ATAGTTTAGCGGCCGCCAGGTGGTTTCTAGCTC
XhoI-CSB-1400-F	CCGCTCGAGCTGAGACCAAAGCAAAAGC
CSB-1493-NotI-R	ATAGTTTAGCGGCCGCGCAGTATTCTGGCTTGAG
XhoI-NLS-CSB-961-F	CCGCTCGAGCCTCCAAAAAAGAAGAGAAAGGTAGGCGGCGGCA GGCTCCTGACTGCG
XhoI-NLS-CSB-1200-F	CCGCTCGAGCCTCCAAAAAAGAAGAGAAAGGTAGGCGGCGGCC TGAGACCAAAGCAAAAGC
CSB-1493-stop-NotI-R	ATAGTTTAGCGGCCGCTTAGCAGTATTCTGGCTTGAG
CSB-AD-10A-F	GCAGCTGCCGCAGCTGCCGCAGCTGCCGCCGTGGAGGGGGC
CSB-AD-10A-R	AGCTGCGGCAGCTGCTGTGGGGAAAT
CSB-AD-6A-up-F	GGAGACTCTGCAGGTGCAGCCTCTGCCTATTTCCCCACAGCAG CTGC
CSB-AD-6A-up-R	GGCTGCACCTGCAGAGTCTCCAGCTGCGGCTGGCCTCATGTCT GACTCC
CSB-AD-6A-down-F	GCGGCCCTGTCTGGAGCTGGTACTGCCTATGCACTGAAGCCTC TGCCCAAGG
CSB-AD-6A-down-R	ACCAGCTCCAGACAGGGCCGCTGCTGCCCCAGCCACGGCGGC AGCTGCGGCAG
CSB-AD-12A-F	GAGGAGGAGGAAGAGGAGGAAGATGACGAGGTGGCTGGGGCA GCAGC
CSB-AD-12A-R	CTCGTCATCTTCCTCCTCTTCCTCCTCCTCTGTGGGGAAATAGG CAGAG

Supplementary Table 2. Sequences of sgRNA oligonucleotides for CRISPR-Cas9 KO

Oligo Name	Sequence (5'-3')
CSB sgRNA4 up F	CACCGATGAATCCTATATAACGAAA
CSB sgRNA4 up R	AAACTTTCGTTATATAGGATTCATC
CSB sgRNA3 down F	CACCGAAGGAGTATCGGTCGGTCC
CSB sgRNA3 down R	AAACGGACCGACCGATACTCCTTC
CSB sgRNA5 up F	CACCGCGTGCCTGGCCTAATTAATC
CSB sgRNA5 up R	AAACGATTAATTAGGCCAGGCACGC
CSB sgRNA8 down F	CACCGAAGTTTAGGAGATGTGTAG
CSB sgRNA8 down R	AAACCTACACATCTCCTAAACTTC
CSB check primer F	CAGCCCCTTGAGTAACTGGG
CSB check primer R	TGCATGCAACTGGCTTTCAC
RAD52 sgRNA L1 up F	CACCGCTAGGCTGGAGTCCGACCAG
RAD52 sgRNA L1 up R	AAACCTGGTCGGACTCCAGCCTAGC
RAD52 sgRNA R1 down F	CACCGACCCACAGCAGACTTTCAGC
RAD52 sgRNA R1 down R	AAACGCTGAAAGTCTGCTGTGGGTC
RAD52 check primer F	AATTCATGTGCCTGGAAAGC
RAD52 check primer R	CCCACGTAGAACTTGCCATT

Supplementary Table 3. Sequences of oligonucleotides for in vitro assays

Oligo Name	Property	Sequence (5'-3')
Oligo 1	ssRNA*	UUAAUUGGUGUGACUAAUCGAAGUUGAUACAUCGAC GUUAUGGUGAUGAU
Oligo 2	ssDNA	ATCATCACCATAACGTCGATGTATCAACTTCGATTAGT CACACCAATTAA
Oligo 3	ssRNA-biotin	UGACUAAUCGAAGUUGAUACAUCGACGUUA-biotin
Oligo 4	ssDNA	TAACGTCGATGTATCAACTTCGATTAGTCA

* Oligo 1 was labeled with maleimide-IR800 probe.
